# Response of *Prochilodus nigricans* to flood pulse variation in the central Amazon

**DOI:** 10.1098/rsos.172232

**Published:** 2018-06-13

**Authors:** Peter B. Bayley, Leandro Castello, Vandick S. Batista, Nidia N. Fabré

**Affiliations:** 1 Department of Fisheries and Wildlife, Oregon State University, Corvallis, OR 97331, USA; 2 Department of Fish & Wildlife Conservation, Virginia Polytechnic Institute and State University, Blacksburg, VA, USA; 3 Federal University of Alagoas, Institute of Biology Science and Health, Cidade Universitária, Maceió, Alagoas, Brazil

**Keywords:** river, floodplain, hydrology, fish, population, habitat

## Abstract

The influence of the flood pulse on fish populations has been posited, but infrequently tested or quantified. Here, we tested the effect of habitat on population size, using *Prochilodus nigricans* as a case study species. Floodplain habitat was based on the littoral zone area occupied by *P. nigricans* to feed. The magnitude of this habitat in each hydrological year, the moving littoral (ML), was expressed as the sum of daily littoral areas during the advancing flood pulse, using satellite-based passive microwave data. Annual population size was estimated by age class, using a dynamic age-structured model (MULTIFAN-CL) based on catches, effort and fish length frequencies from the Manaus-based fishery over 12.75 years. The principal null hypothesis was that the ML, using three lag times, had no effect on population size of a single age class of *P. nigricans*. The population size at 29 months of age was positively related (*p* = 0.00030) to floodplain habitat (ML) earlier in the same year, when the fish were 21–27 months old. The result implies a density-dependent relationship for the population with respect to its feeding habitat. Potential mechanisms governed by flood pulse variation and habitat quality for this and other species using floodplain habitats are discussed.

## 1. Introduction

How does a fish population respond to hydrological variation in a river floodplain? This question can be indirectly addressed by analysing a time series of fishery yield as a function of hydrological variables that are thought to increase or decrease yield. This approach has had some success [1]. Van Zalinge *et al*. [2] and de Graaf [3] reported positive correlations of yield with flooding indices occurring earlier in the same year, from the Tonlé Sap bag fishery in the Mekong and from a Bangladesh floodplain fishery, respectively. Welcomme [4] reported greatest correlations from varying proportions of a flooding index for one and two preceding years among three African floodplains, but the index was correlated negatively to a drawdown index. Smolders *et al*. [5] reported greatest correlation of *Prochilodus* yields with a mean flooding index over the previous 3 years. Castello *et al*. [6] found in the lower Amazon best correlations between yields of various trophic groups and a flooding index 2 or 3 years previously, along with fishing effort and a drawdown index of 2 or 3 years previously. Quiros & Cuch [7] reported *Prochilodus* yields correlated with high water 4 or 5 years previously, while they were also negatively correlated with drawdown in recent years.

However, this approach has also failed to detect anticipated correlations between hydrology and fish yields, even over long periods or with liberal multi-testing. Risotto & Turner [8] analysed yields of seven species over 23 years from the lower Mississippi River basin, and did not find any hydrological relationship for any species while accounting for effort, temperature and regional effects. From the database analysed here, annual *P. nigricans* yield in the central Amazon was uncorrelated with a flooding or drawdown index of 1, 2 or 3 previous years, with or without effort being included. Addressing the question at the beginning of this section by using yield as a proxy for population size or production is understandable given the availability of data, but there are reasons why the proxy is inappropriate for such purposes. Annual fish yields typically contain several age classes whose structure varies due to strong and weak year classes that may or may not be driven by flood pulse variability. Even accounting for fishing effort, annual fish yield may fail to reflect hydrological effects because such effects in a given year on fish of a given age would be reflected in several future yields. Therefore, each annual yield contains contributions from age classes that would be affected by hydrological conditions during several preceding years, diluting the response. This can partly explain why empirical results often do not identify specific or consistent lag periods, except when short-lived species dominate the fishery (see citations above). Despite these issues, fish yields often respond positively to some index of prior flooding, or negatively to a drawdown factor, but ascribing ecological or fishery mechanisms is difficult without additional information [1]. Investigating a single year class can produce a significant and more meaningful relationship. For example, the catch per unit effort of young-of-the-year fish has been found to respond positively to flooding among several migratory species, including *P. lineatus*, in the Upper Paraná River [9].

The general question addressed in this paper is whether the amount of floodplain habitat affects population size. A multi-age population estimate would incur the same problems of attribution as multi-age yields, but in our analysis we estimated annual population by age class. The null hypothesis we tested, using various lag times, is that the magnitude of floodplain habitat availability, as influenced by the annual hydrological regime, has no effect on the population size of a given age class of *P. nigricans*. Our alternative hypothesis is that the population of a given age class increases with available habitat. We also tested for the effect of drawdown during the low water season. This species was selected because: (a) its life history of main channel spawning and floodplain growth is characteristic of many species that collectively dominate biomass or yields in tropical river floodplains [1] and (b) it is a common and preferred species in the Amazon, enabling the fishery to serve as an instrument to sample the population.

**Figure 1.**
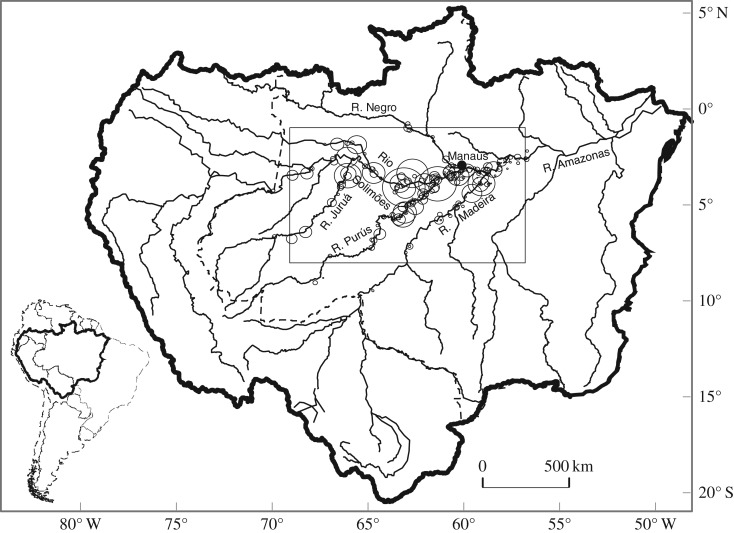
Location of the study area (rectangle) in the Amazon basin (thick line). Circles show relative sizes of cumulative catches of *P. nigricans* for each location that supplied Manaus market (solid circle) between 1993 and 2006.

We used fishery data to estimate annual population size by age class and a combination of field samples and remote-sensing data to derive estimates of the amount of floodplain habitat available to *P. nigricans* for each hydrological year. Our study is organized as follows: first we describe *Prochilodus* life history, including habitat use and growth of *P. nigricans* in the study area (figure 1). This information is then combined with hydrological and remote-sensing data to provide annual estimates of the magnitude of the moving littoral (ML) habitat [10,11] preferred by this species when occupying the floodplain. Then, following a description of the fishery, we apply an age-structured model to the fishery data in order to derive estimates of population size at age by year. We compare these estimates to the ML. Background and methods are included as appropriate in the following four sections.

## 2. *Prochilodus* life history

**Figure 2.**
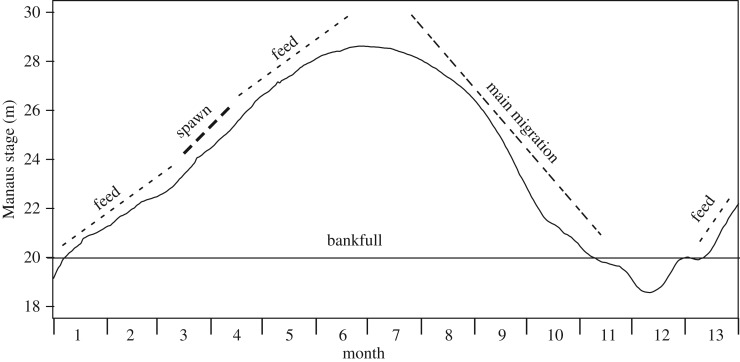
Simplified lifecycle of *P. nigricans* during a typical hydrological cycle. ‘Bankfull’ is an approximate level corresponding to a transition from most water-occupying channels and permanent lakes to an acceleration of inundation of the floodplain. ‘Feed’ represents feeding in the ML of the floodplain. In March–April a spawning migration occurs from the floodplain to a white-water river and then back to the floodplain, via channels. ‘Main migration’ (piracema) involves an upstream migration in a white-water river.

*Prochilodus* spp. are found in the major basins of South America and are frequently dominant in biomass [12,13] and yield [7,14]. The following synopsis of their common life history draws from [15–20], and personal observations and dialogue with Amazon fishers by all the authors. *Prochilodus* spp. are potamodromous, with various migration patterns, and are highly fecund total spawners, corresponding to r-selected species. In smaller basins or tributaries with a single annual flood pulse they migrate upstream in the main channel (known in Brazil as ‘piracema’) during low water to spawn at rising water, and then move downstream to enter floodplains to feed. In the central Amazon, the main migration occurs in white-water rivers during falling water (July–October) (figure 2). After the water has begun rising (November–January), they seek out white-water-influenced floodplain (várzea) habitats to feed. They return to the river (March–April) via channels to spawn and then return to feed again in the floodplain (independent interviews with fishers by N. Fabré and P. Bayley). In all migrations they are often accompanied or followed by immature fish. Downstream migration is difficult to observe because the schools do not surface as in the main migration, but has been proved in non-Amazon rivers where tagging has provided returns [15,17].

**Figure 3.**
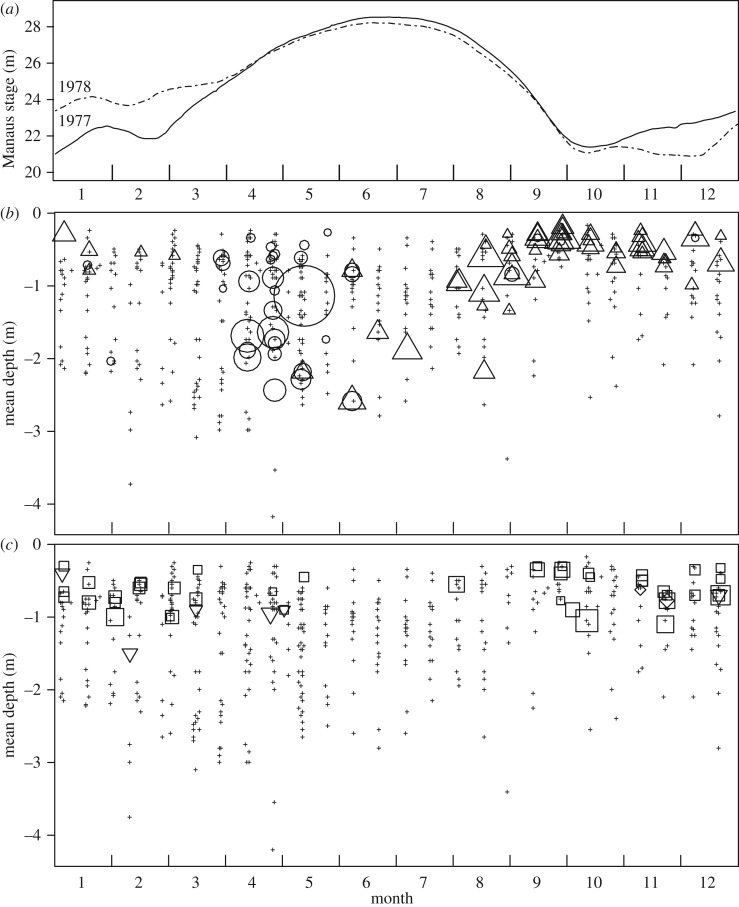
The presence/absence of *P. nigricans* by water depth and month, based on 623 daytime 25 m seine samples among three floodplains in the central Amazon from February 1977 to May 1979 (*b*,*c*) [23], and Manaus stages for 1977 and 1978 (*a*). In (*b*), circles are for fish lengths greater than or equal to 1.5 and less than 10 cm, triangles greater than or equal to 10 and less than 17 cm; in (*c*) squares are for fish lengths greater than or equal to 17 and less than 25.5 cm (Age1 in text), diamonds greater than or equal to 25.5 and less than 30.5 cm (Age2 in text), and inverted triangles greater than or equal to 30.5 cm (Age3+ in text). In (*b*) and (*c*), ‘+’ denotes the sample with zero *P. nigricans* catch; other symbol areas are proportional to abundance density (catch data corrected for catchability [24]).

**Figure 4.**
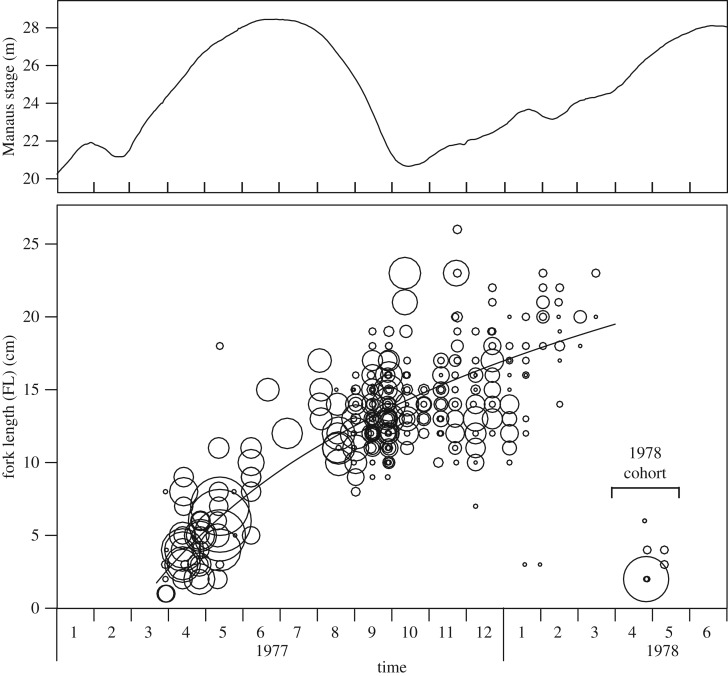
Growth of juvenile *P. nigricans* in the central Amazon floodplain from 1279 individuals caught in 160 25 m seine samples [23] and concurrent stage data. Symbol areas proportional to fish density (corrected for catchability [24]). Fit for the 1977 cohort based on linear regression on log(time) weighted by fish density.

Pelagic eggs and larvae, developing rapidly, drift with the current and spread into floodplains as the water rises. In the Paraná River young post-larval fish 7–14 mm long feed on zooplankton and change to a finer diet of rotifers, protozoa and detritus at 14–33 mm [21]. The final diet change to pure detritus (iliophagous) may occur when fatty acid composition decreases for fish between 38 and 75 mm [22]. In the central Amazon floodplain, the post-larval and young juvenile fish were found in the ML from 0 to 3 m depth (figure 3*b*) corresponding to up to about 100 m from the current shoreline. Older, iliophagous fish feed on the bottom or from macrophyte leaves, and tend to prefer an even more shallow part of the ML, mostly within the 0–1 m depth range (figure 3*c*), which corresponds to about less than or equal to 50 m from the current shoreline. A distinct species group excluding *P. nigricans* prefers pelagic habitats farther offshore [25]. Growth during the first year is rapid and spawning in March–April is apparent (figure 4), as was also observed further upstream [20].

**Figure 5.**
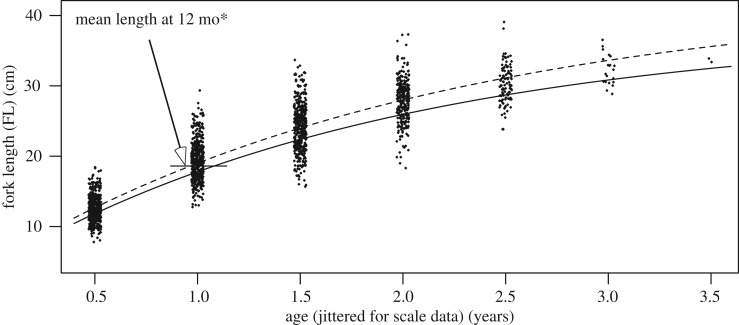
Comparison of growth rate estimates of *P. nigricans* in the central Amazon. Dots are back-calculated fork lengths from scale growth checks from 1673 measurements from 458 individuals sampled from Manaus market and floodplain lakes in central Amazon [26]. Whole number ‘age’ values correspond to growth checks during spawning migration, while half-year ‘age’ values correspond to growth checks during main migration. Plots are ‘jittered’ on the age scale for visibility [27]. Dashed line is von Bertalanffy fit to scale data (derived from [26] using [27]). Solid line is MULTIFAN-CL's von Bertalanffy fit to length frequency data (see text). *Arrow shows the estimate of the mean length at 12 months of age of the 1978 cohort (figure 4).

Size at age has been estimated from scales of *P. nigricans* (40–396 mm long) collected from the Manaus fishery and from locations in our study area throughout the hydrological cycle [26]. While females grow slightly faster than males, the difference is small compared to the range of individual size at age. Here, we report results for sexes combined. Braga de Oliveira [26] interpreted two growth checks per year, about six months apart. The first was associated with the cessation of feeding during the spawning and associated lateral migration in March–April (figure 2), and the second was associated with the cessation of feeding during the main migration upstream in July–November (figure 2). *Prochilodus nigricans* are mature at 2 years of age, but immature fish are also caught during both migrations, in common with migratory Characiformes and Cypriniformes worldwide [1]. Projected scale-check data [26] and a von Bertalanffy model fit are shown in figure 5 (dashed line). Four other *Prochilodus* populations (including three analysed by sex) that produced good von Bertalanffy fits indicated a total length at the end of the first year of between 19.3 and 23.2 cm based on scales, operculae or length frequencies [28].

## 3. The moving littoral

**Figure 6.**
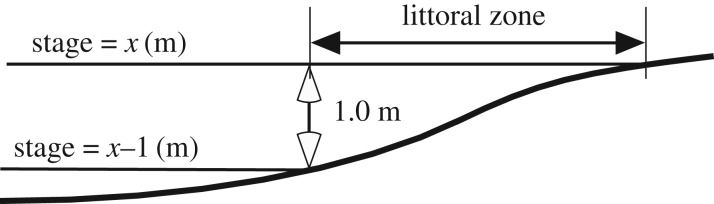
Schematic of a cross section of a floodplain littoral zone (exaggerated vertically). The habitat area of the littoral zone from 0 to 1.0 m depth in a region is calculated as the difference between the estimated total flooded area (figure 7) at stage *x*, time *t* (*A_x_*_,*t*_) and the corresponding area at stage *x* − 1 m (*A_x_*_−1,*t*_). The ML is the sum of littoral surface areas in a defined period during a hydrological season (see text).

**Figure 7.**
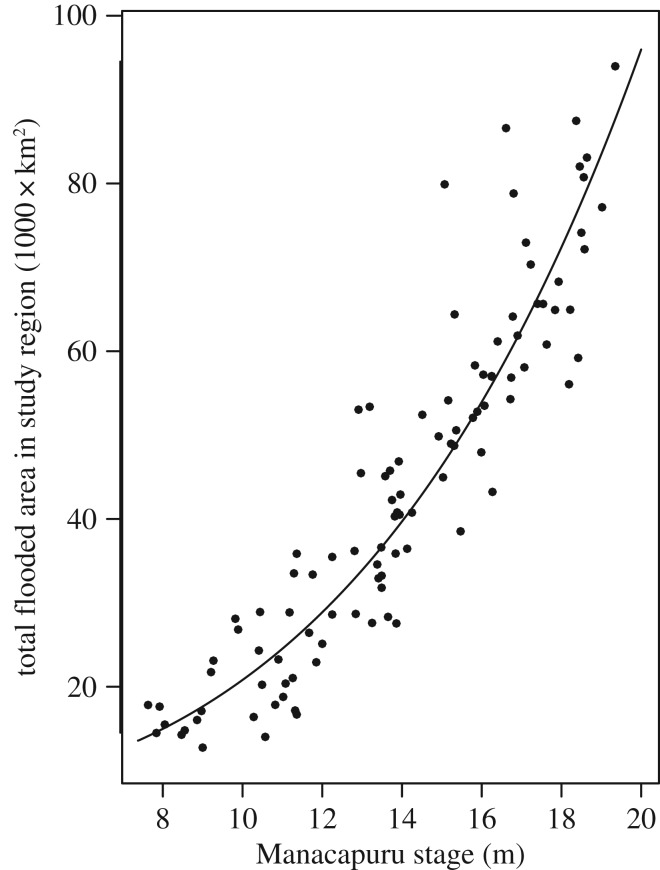
Passive microwave SMMR (Scanning Multichannel Microwave Radiometer) estimates of the total flooded area from 103 monthly measurements from December 1978 to August 1987 (using data for Reaches 2 through 9 [29]) corresponding to the study region (figure 1) versus river stage at Manacapuru (on the R. Solimões 105 km upstream of the Rio Negro mouth). The fitted line is a second-order polynomial: flooded area (km^2^) = exp(36.70–9.40*log(Stage(cm)) + 0.800*(log(Stage(cm)))^2^); *R*^2^ = 0.86. A minimum area of 8870 km^2^ is predicted at a stage of 3.6 m. (Manacapuru stage data are highly correlated to those at Manaus (*R*^2^ = 0.99), and predictions are interchangeable at the scales used here. Mean difference between Manaus and Manacapuru stages = 9.48 m.)

**Figure 8.**
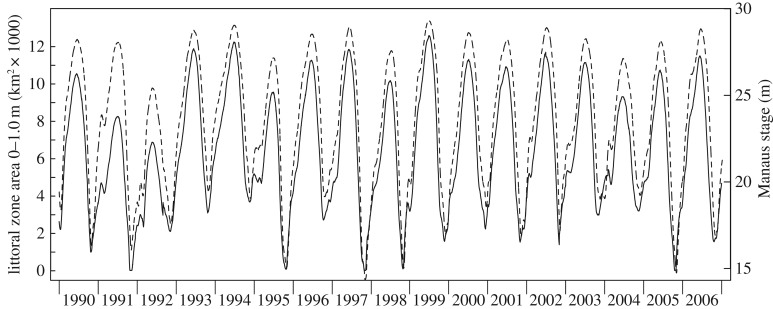
Daily littoral zone area (0–1 m depth) in the study area (solid line) and Manaus stage (dashed line).

The early life, juvenile and adult stages of *P. nigricans* during the inundation period of the flood pulse depend largely on the quantity and quality of inundated floodplain habitat available. The ML concept [10,11] posits that a productive inshore zone traverses the floodplain during the seasonal advance and retreat of the flood pulse. Here, we quantify the ML concept. At a given stage and time the areal extent of the littoral zone is estimated as the difference between the total flooded area at a given stage and the total flooded area corresponding to the stage when it is a given depth lower than the current stage (figure 6) in the region of interest. The zone occupied by the age groups of *P. nigricans* analysed is shown in figure 3*c*, which indicates a depth range of 0–1 m. Currently the best available estimates of total flooded area that can be predicted from the stage in our study area are from an interpretation of passive microwave (SMMR) data from the Nimbus-7 satellite [29]. Their data corresponding to our study region (figure 1) were related to stage (figure 7), which we used to estimate daily littoral areas for the time period of interest (figure 8).

**Figure 9.**
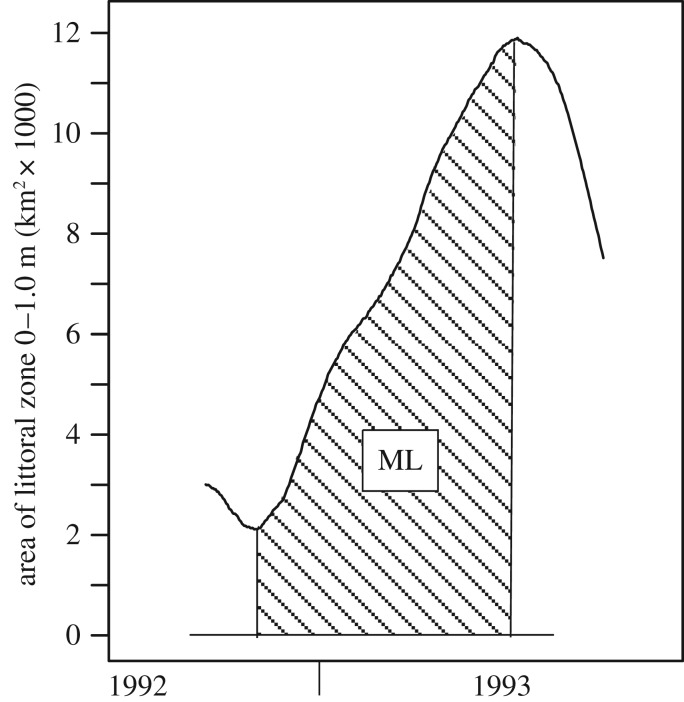
Graphical example of a moving littoral calculation during the advancing flood pulse, ML = ∑(*A_x_*_,*t*_ − *A_x_*_−1,*t*_), shown as area under the curve for estimates of daily littoral zone areas (0–1.0 m) for hydrological year 1993.

The ML is a dynamic extension of the littoral zone, and its annual magnitude is quantified as the sum of daily littoral areas over a defined hydrological season: ML = ∑(*A_x_*_,*t*_ − *A_x_*_−1,*t*_), where *A_x_*_,*t*_ and *A_x_*_−1,*t*_ are total flooded surface areas on day *t* and stage *x* and *x* − 1, respectively, that are summed over consecutive days in a hydrological season. The ML therefore has a dimension L^2^T. Essentially, the ML is a time-weighted extension of the littoral area that reflects the annual change in floodplain habitat. However, the advancing and retreating periods are very different in terms of inshore habitat [10], and higher growth increments have been shown for juveniles of several species, including *P. nigricans*, during the advancing stage [30]. Therefore, we computed the ML for the advancing flood pulse (minimum to maximum stage) (figure 9) and for a drawdown period following the maximum stage.

A second property that might influence population size is the rate of rise of the hydrograph (RR), reflecting the rate of advance of the ML. This has been proposed as a factor to compare river-floodplain systems [11], and for explaining fish growth differences within hydrological seasons [30], but it has not been used for year-to-year effects within a system. We estimated RR by calculating the difference between the minimum and subsequent maximum Manaus stage divided by the number of days elapsed. The ML and RR data are compared with population size estimates in §7.

## 4. The Manaus fishery

Data from the Manaus fishery were used to estimate abundance of *P. nigricans* by age class, in order to relate to annual estimates of ML magnitude. The Manaus city market is a major transit area for commercial fisheries in the central Amazon (figure 1). Monitoring of catch, effort and fish length frequencies was conducted at the Manaus market by trained assistants [31]. The returning boat operator was interviewed to obtain the fish catch by weight, dates spent fishing, number of fishers, fishing gear used and fishing location. No taxes were levied regarding quantities caught, and the fisher's privacy was respected regarding publication of individual names, boats, or associated catch and location. Records from 30 702 trips were obtained from 1 July 1993 to 31 March 2006. A portion of these was periodically sampled for length frequencies, comprising 1606 samples in which fork lengths of 21 279 individuals of *P. nigricans* were measured to the nearest centimetre down.

For our analysis, we needed totals for fishing effort by quarter and by ‘fishery’, defined by gear type (for details of how this information was derived, see the electronic supplementary material, Manaus market data processing).

During the 12-year nine-month period monitored, the total estimated multispecies catch was 311 192 t, of which 45 959 t (15%) was *P. nigricans*, a yield exceeded only by two taxa, ‘jaraqui’ (mostly *Semaprochilodus insignis*) and ‘pacu’ (mostly *Mylossoma duriventris*). Two gear types, lampara seine and gill nets, accounted for most of the yield (table 1). Only 0.08% of the total *P. nigricans* yield was accounted for by trips that did not use either of these gear types, and the 549 trips involved were excluded from the analysis.

**Table 1.** Yields (t) from the three dominant fisheries exploiting *P. nigricans.*

yield (t)	total (%)	lampara (fishery 1)	gill net (fishery 2)	lampara and gill nets (fishery 3)
quarter				
Jan–Mar	8033 (17%)	5026	577	2430
Apr–Jun	7225 (16%)	5620	144	1462
Jul–Aug	17 856 (39%)	14 454	238	3165
Sep–Dec	12 762 (28%)	9652	687	2423
totals	45 877	34 751 (75.7%)	1647 (3.6%)	9479 (20.7%)

## 5. Analysis of fishery data

The Manaus market data were analysed using MULTIFAN-CL (MFCL), which is appropriate for estimating population abundance by age class because it has a well-established statistical pedigree as a length-based, age-structured dynamic model [32–34]. Although MFCL can analyse different regions with hypothesized movement of the stock among them, neither the fisheries data nor the biological information were sufficient to warrant a multi-region approach. The area subject to the Manaus fishery for *P. nigricans* (figure 1) is centred on the central Solimões-Amazonas River floodplain, which merges with the floodplains dominating the lower reaches of other white-water tributaries, such as the Purus and Madeira rivers. Given the large size of this region and the centrality of most of the floodplains, any net migration to or from surrounding areas is considered small in relation to the stock modelled. Conversely, our defined *P. nigricans* stock in this region may well be a meta-population in the form of populations with varying degrees of overlap and intermixing. Therefore, the derived parameters can be regarded as averages representing one or more populations.

For details of MULTIFAN-CL and how the results were derived, see the electronic supplementary material, MULTIFAN-CL analysis.

## 6. Fishery model outputs

Four age classes were determined, and are referred to as Age1, Age2, Age3 and Age4. An analysis presuming five age classes did not result in a significant decrease in the objective function (at *p* = 0.05).

**Figure 10.**
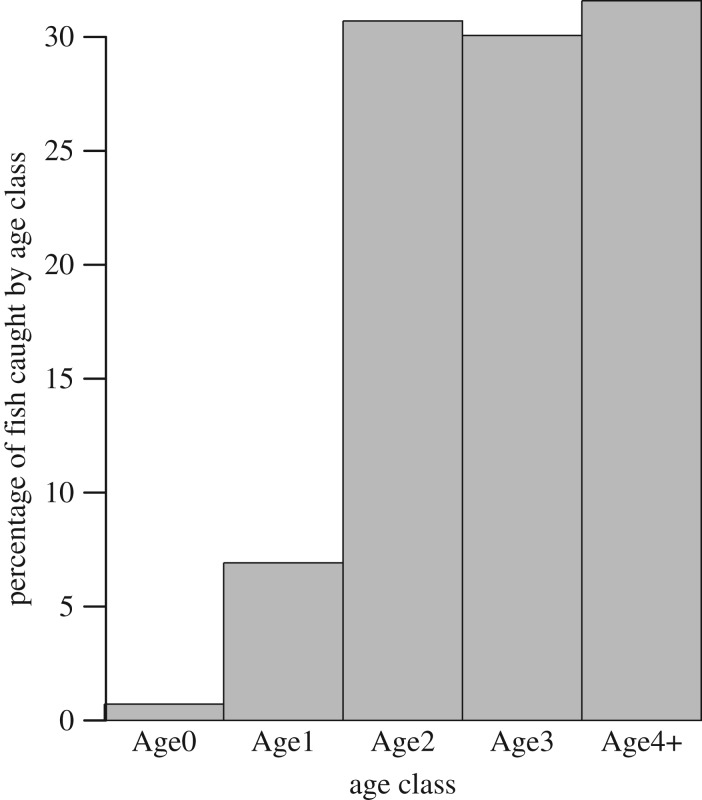
Percentage of numbers of *P. nigrican*s caught by age class from all fisheries and years. Age classes Age1 through Age4+ were derived from MFCL [34], with size at age calculated from MFCL's von Bertalanffy fit (figure 5, solid line). Age classes correspond to fork lengths of 17–25.4 cm (Age1, 1 year five months old), 25.5–30.5 cm (Age2, 2 years five months); 30.6–33.6 cm (Age3, 3 years five months), and greater than or equal to 33.7 cm (Age4+, greater than or equal to 4 years five months). The ‘Age0’ age class of less than 17 cm was not detected by MFCL.

MFCL requires a date approximating when recruitment occurs in order to estimate and express results by age class each year for each iteration. Most *P. nigricans* are recruited to the fishery at Age2, whereas a much smaller number of Age1 fish were caught (figure 10). Most Age2 fish were caught during the third quarter, July–August, a period of maximum drawdown rate when they are departing the floodplain and entering the white-water rivers to migrate upstream. Over 75% of Age2 fish were caught by lampara seine (fishery 1) during this period. The lowest objective function value was obtained for a recruitment date of 1 September during this period.

The estimation of a realistic average growth rate is obviously critical. We allowed MFCL to derive a fit by setting wide ranges of priors (size-at-age for Age1: 13–38 cm, Age 4: 30–52 cm; von Bertalanffy's *K*: 0.30–0.53; s.d. of length-at-age: 3–8 cm) to compare model output with the independent scale-based data described above, because the scale samples were not subsamples of the monitored fishery, and collection dates (1989–1993) only partially overlapped those of the fishery (1993–2006). The final von Bertalanffy fit predicted that mean lengths on 1 September were 21.7, 28.3, 32.3, 34.7 cm for Age1 though Age4, respectively, and *K* = 0.512. At a mean birth date of 1 April, these means corresponded to ages of 17, 29, 41 and 53 months, respectively. The resulting age-at-length function FL*_t_* = *L*_∞_(1 − exp(−0.512(*t* + 0.223))), where FL*_t_* = fork length at age *t* and *L*_∞_ = 38.2 cm, is shown in figure 5 (solid line). The comparison with scale data indicates a reasonably close correspondence (figure 5), given the variation of projected lengths from scale marks, and the length-at-age s.d. of 3.53 cm estimated by MFCL.

Natural mortality, *M*, was estimated by the model as 0.34 yr^−1^. As this implicitly includes all forms of mortality other than that of the Manaus fishery, the effects of other, local markets and subsistence fisheries are included in *M* by default. Mean estimates of fishing mortality, *F*, for Age1, Age2, Age3 and Age4 were 0.12, 1.02, 1.68 and 1.73 yr^−1^, respectively. Selectivity was estimated by age class and fishery (electronic supplementary material, table S1). The population size estimates from the MFCL run with the lowest objective function are presented in table 2.

**Table 2.** Population size of *P. nigricans* estimated by MFCL by age class for 1 September in each year (in billions).

year	Age1	Age2	Age3	Age4+
1992	0.0083	0.0087	4.71	1.07
1993	33.0	0.0054	0.0029	0.0970
1994	2.49	16.1	0.00017	0.00013
1995	34.6	1.67	6.87	0.000015
1996	4.32	18.5	0.0894	0.185
1997	1.66	2.88	7.59	0.0519
1998	21.4	1.13	1.40	3.583
1999	8.98	10.4	0.032	0.100
2000	9.33	6.01	4.39	0.0282
2001	13.0	6.30	2.68	1.76
2002	12.7	8.58	2.34	1.46
2003	5.40	8.71	4.23	1.63
2004	9.69	3.63	3.68	2.05
2005	93.1	6.53	1.64	2.31

MFCL provides an estimate of annual spawning biomass, given an input of proportion of mature females by age group and assuming a consistent sex ratio. There was an estimated 19-fold variation in spawning biomass, but there was no indication of a stock–recruitment relationship when it was compared with the fully recruited Age2 population estimates 2 years later.

## 7. Hydrological effects on abundance

To test whether the magnitude of the ML (figure 9), as influenced by hydrology during rising water, has no effect on the population size of a given age class of *P. nigricans*, we had to define the period in which the involved ecological processes take place. Given our limited biological knowledge, we did not know at what age(s) the fish were likely to be affected by ML, so independent multiple tests were required for different lag times. However, the number of independent tests was limited because there were strong correlations of population size among age classes of corresponding cohorts (table 2). For example, a strong cohort, such as the large value for Age1 in 1995, maintained its dominance as Age2 in 1996 and Age3 in 1997. Age1 and Age2 values for corresponding cohorts 1 year later were strongly correlated (*R*^2^ = 0.975, *N* = 13). The small unexplained variance reflects differences in estimated total mortality mainly due to fishing effort variation. Similarly, Age2 and 1-year lagged Age3 values were strongly correlated (*R*^2^ = 0.986).

These strong correlations among the age class response variables limited the number of available independent tests, because each member of a correlated pair of responses would be tested with the same set of ML values. Also, Age3 and older population estimates become increasingly dependent on the cumulative effect of total mortality, dominated by fishing mortality. Therefore, we considered all possible tests for Age1 and Age2 responses to ML for up to three lag periods (table 3). The test of Age2 as a function of ML during the previous year (lag 1, test 2–1, table 3) was not independent from the test of Age1 on the same ML values during the same year (lag 0, test 1–0). Similarly, an Age2 test on ML 2 years previously (lag 2, test 2–2) was not independent of that of Age1 on the same ML values the previous year (lag 1, test 1–1). Regarding independence of the explanatory variable, ML, there were no correlations between lag 0 and lag 1 (*p* > 0.9) or between lag 1 and lag 2 (*p* > 0.34). Consequently, three among the five test options in table 3 were available as independent tests.

**Table 3.** Five options to test effect of the moving littoral (ML) on population size of age classes Age2 and Age1. n.a., not applicable.

response	lag (years)^a^	0	1	2
Age2 (29 months old)		2–0	2–1*	2–2**
Age1 (17 months old)		1–0*	1–1**	n.a.

^a^lag = 0 compares response to the ML earlier in the same year, when Age2 fish were 21–27 months old and Age1 fish were 9–15 months old; lag = 1 compares response to the ML 1 year previously, when Age2 fish were 9–15 months old and Age1 fish were 0–3 months old; lag = 2 compares response to the ML 2 years previously, when Age2 fish were 0–3 months old.

* and ** denote pairs of tests that are not independent (see text).

The reliability of MFCL population outputs depends on which age classes were most intensively sampled by the fishery. Age1 fish recruitment was minimal (figure 10). The average fishing mortality (*F*) compared to the total mortality (*Z*) was 75% for Age2, while the proportion for Age1 was only 25%. To predict Age1, MFCL was effectively projecting back from Age2 with an assumption that 75% of the total mortality was a constant natural mortality rate that, in common with most fisheries, cannot be independently estimated. Also, the interpretation of fishing effort for Age1 using the catch equations is questionable due to discarding of *P. nigricans* in the Age1 size range due to lower market value, for which evidence in the Amazon exists [35].

**Figure 11.**
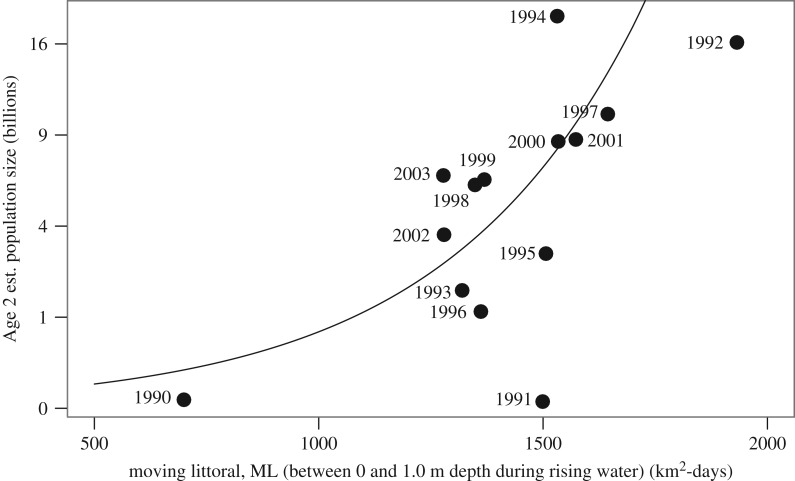
Population size (estimated by MFCL) for ‘Age2’ (= 29 months of age) versus the moving littoral, ML (0–1.0 m depth) during the rising water period earlier in the same hydrological year. Curve is a negative binomial fit [27,36] with a count response in 1000 s of fish. Symbol labels indicate birth year of each cohort. Model prediction: (Age2 popn.)/1000 = exp(8.871 + 0.00459*ML); *t*-value for ML coefficient = 3.89 (*p* = 0.00010). Fitted dispersion coefficient (Theta) = 0.75 (s.e. = 0.24). Residual and null deviances were 20.84 and 16.76, respectively.

**Figure 12.**
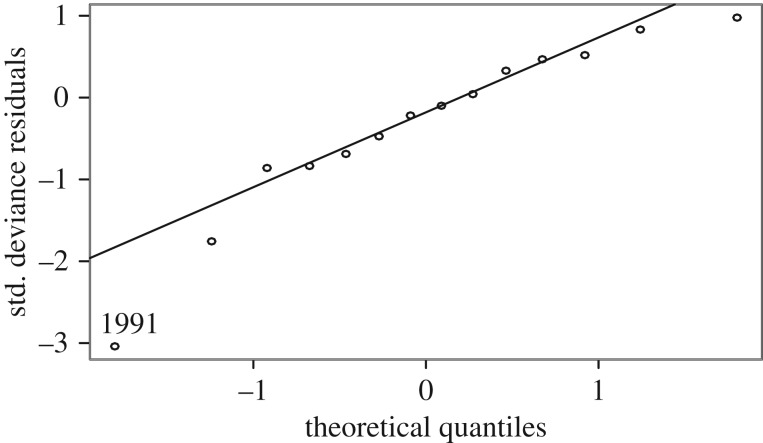
Normal Q-Q plot for deviance residuals from the model shown in figure 11 [27].

Therefore, we used the three independent tests for Age2 (table 3). Regarding the first test of Age2 with the ML earlier the same year (2–0), the best model in terms of residuals and significance for the ML was obtained from a negative binomial model (figure 11) with a type I error of *p* = 0.00010 for the ML coefficient. Residuals were well-behaved except for that of birth year 1991 (figure 12). A simple linear regression (*p* = 0.014) was unrealistic because it predicted negative values for Age2 population size when the ML was small.

The remaining independent tests 2–1 and 2–2 for lags of 1 and 2 years, respectively, failed to converge, but their linear models provided no indication of a relationship (*p* = 0.81 and 0.85, respectively). Considering that the mean birth date of April 1 occurs about halfway through the advancing ML period, test 2–2 was repeated for an ML calculated from 1 April to peak flood, but the linear result was again not significant (*p* = 0.60).

The second property of the ML, rate of rise of water level (RR), was not correlated with ML (*p* = 0.84), and when included as an additional explanatory variable in test 2–0, its coefficient was not significant (*p* = 0.64).

Another potential effect on population size is the extent of the drawdown period, commonly defined as the area under the hydrograph (figure 8, dashed line) below the estimated stage at ‘bankfull’ (figure 2). We computed drawdown, *D*, as the sum of the daily differences between bankfull and the stage for each hydrological season when the stage was below bankfull. The usual implication of a negative effect of drawdown is that it would increase mortality during that period. While our fisheries model was based on a constant natural mortality estimate, deviations from Age2 predictions could potentially be explained by a previous drawdown. *D* was not correlated to ML. When *D* was added as a second predictor in the Age2 models (table 3), it was not remotely significant for any of the three lags corresponding to those used for ML, even though *D* had a 41-fold variation.

There are other potential indices of floodplain habitat that are correlated to varying degrees with ML at a 0–1.0 m depth. Broader (0–1.5 m) and narrower (0–0.5 m) interpretations of littoral habitat (figure 3*c*) were tested, and both gave identical results at two significant digits. The ML so far has been calculated for the advancing ML (figure 9), because *P. nigricans* is known to leave the floodplain and join upstream migrations during drawdown. However, some do stay behind during July–September (figure 3*c*). Consequently, the magnitude of the retreating moving littoral (MLr) that begins at the maximum stage and ends at the date of population estimation, 1 September, was computed. MLr was uncorrelated with ML (*p* = 0.086). When included as an additional explanatory variable in test 2–0, its coefficient was not significant (*p* = 0.42), while that of the ML maintained high significance (*p* = 0.0010). Therefore, changes in the retreating ML, which varied 1.76-fold, had no significant effect on the Age2 population in the same year.

Dudley's [4,37] FI index of flooding is the sum of the weekly stages from bankfull to the maximum stage. This was calculated on a daily basis from Manaus stages and tested in lieu of the ML. The negative binomial model at lag 0 produced a good fit at *p* = 0.00053. For comparison, our most significant relationship was found for an equivalent ML magnitude computed from bankfull to the maximum level, which produced a type I error (*p* = 0.000014) 38 times smaller. However, all these alternatives depend on a subjective estimate of the stage at bankfull.

The best relationship with Age2 was indicated by the significance of the ML coefficient, computed from the minimum to the maximum stage, at lag 0 (figure 11), while longer lags indicated no significance. Because three tests were used to address the hypothesis, a Bonferroni correction for a more realistic type I error estimation was applied, producing an adjusted *p* of 0.00030. In conclusion, the magnitude of the ML at 0–1.0 m depth in any given year is a strong predictor of the Age2 population size later in the same year.

## 8. Discussion and conclusion

Our principal result demonstrates that the *P. nigricans* population at 29 months of age is positively related to the available floodplain habitat (ML) earlier in the same year, when the fish were 21–27 months old. This implies that some density-dependent process may be occurring during the season when they are feeding.

The relationship also suggests that the response accelerates as the ML increases. In years when the ML is small, a given increase (such as ML = 900 → 1200 km^2^ d) predicts an increase in the Age2 population of 1.3 billion, whereas a similar increment in the ML from 1200 → 1500 corresponds to a 5.2 billion increase (figure 11). If this acceleration is real, as opposed to a linear increase, a stronger density-dependent process is implied at higher densities in years when the ML is larger, which occurs when floods are higher and/or of longer duration (figure 9). *Prochilodus nigricans* feeds on fine detritus, but less-nutritious C_4_ grasses dominate the macrophyte assemblage in the floodplain. Oliveira *et al*. [38] found that carbon isotope values (∂^13^C) from *P. nigricans* were associated with those of C_3_ plants in the central Amazon. A preliminary analysis of the samples [23] (figure 3) indicated that the average biomass density of *P. nigricans* was four times greater from samples where the C_3_ plant, *Oryza*, was present than when it was absent. *Oryza* spp. are more common in the ML at mid to high water levels [39]. The implication here is that food quality of the ML may increase at higher levels, from say April to June, permitting higher *P. nigricans* densities. This is consistent with the aforementioned accelerated population increase for high ML values (figure 11). Further study, including other detritivores and other C_3_ plants and their distribution during different hydrological cycles, is called for.

Why were there no significant relationships for Age2 abundance and ML magnitude during its previous year (9–15 months old) or its birth year (0–3 months old)? Oliveira *et al*. [9] found a strong flooding effect on young-of-the-year, in addition to a flood timing effect, in the dam-influenced Upper Paraná river. Our findings do not exclude other mechanisms that may regulate *Prochilodus* when their diet is dominated by zooplankton during their first few weeks of life. The larvae are pelagic and spread over larger areas, in contrast with post-larval fish that appear to be restricted to the ML less than 3 m deep (figure 3*b*). When seasonal habitat types within and beyond the ML are quantified using recent ALOS/PALSAR ScanSAR satellite data [40] (L. L. Hess 2017, personal communication), the surface areas of non-forest zones, where plankton is likely to be more productive, could be estimated and compared with population sizes corresponding to birth years.

Another reason why the ML for the previous year or the birth year was not significant may be due to Age2 populations not reflecting the relative population sizes for their younger cohorts at the time of the corresponding ML values, which occur more than 1 year earlier. As stated above, Age1 estimates depend strongly on the assumption of constant natural mortality, which averaged 75% of the total mortality. This resulted in strong correlations between Age2 and Age1 populations 1 year earlier, affecting the independence of tests (table 3). These correlations would be weaker if natural mortality varied from year to year, with a much stronger influence on Age1, because Age2 estimates are largely determined by known fishing catch and effort. This might explain the density-dependence implication of those surviving to Age2. After projecting back from fully recruited Age2 population sizes (table 2) using different, hypothetical year-specific natural mortality rates, the latter was found to require a variation of more than an order of magnitude to produce Age1 estimates that might significantly correlate with ML.

The use of passive microwave data (SMMR) [29] to estimate flooded area should be regarded as provisional because less-biased predictions are anticipated from Synthetic Aperture Radar (SAR) information from the JERS-1 satellite [41], which is due to be processed with recent digital elevation (DEM) information. A preliminary comparison between SMMR and JERS high- and low-water estimates for 12 Amazon main stem reaches used by Sippel *et al.* [29] (L. L. Hess 2017, personal communication) indicates that SMMR values consistently underestimated those of JERS by about 20–35%. While we anticipate that the absolute ML estimates for our study area, modelled on JERS estimates based on river stage (as were SMMR values in figure 7), will be increased, the variation from year-to-year is expected to be similar.

What are the implications regarding other species and river-floodplain fisheries? The use of total yield as a response to hydrological change is limited in those fisheries that exploit a variety of age classes with varying year-class strength [42], even when the purpose is to predict yield. In our study, direct prediction of *P. nigricans* yield using hydrological predictors failed. However, the success in explaining population size according to habitat as a function of previous hydrology allows us to predict future yields as a function of different ML scenarios using the catch equations.

Applying specific biological information to help define floodplain habitat quantitatively has here produced a better predictor for population size for the first fully recruited age group than previous indices of hydrology, and this approach provides a basis for exploring issues of habitat quality. The strong relation between habitat size and population for a major species reported here gives some credence to the assumption of density dependence when developing simulation models such as that by Halls & Welcomme [43]. Their model assumed density dependence at the larval stage, which we have not disproved. The life history of *P. nigricans* is characteristic of many river-floodplain species that spawn and migrate in the main channel and grow in the floodplain. While most derive their energy from primary productivity in the floodplain [10], their use of the large variety of floodplain and riverine habitats can differ. For example, an offshore group of species uses the littoral zone at depths greater than shown here for *P. nigricans* [25], calling for a different version of the ML to describe habitat magnitude.

Extending this knowledge base using individual species in other systems remains a challenge, however. The combination of ecological, fishery and interpreted satellite information in the central Amazon that we have been able to use is not replicated in many other river floodplains, and extrapolation of findings needs to be heavily qualified because degrees of alteration of hydrology and natural vegetation vary considerably. However, comparison of the magnitude of the ML among systems, while accounting for habitat quality and the timing of flood pulses, is feasible with current technology and adequate ground truthing. The flood pulse concept provides a general guide for a very complex system, and while some broad relationships may show consistency across systems [1,44], fine-tuning for individual species or guilds may become more system-specific.

## Supplementary Material

Supplementary Material: Response of Prochilodus nigricans to flood pulse variation in the central Amazon

## Data accessibility

Data are available from the Dryad Digital Repository: https://dx.doi.org/10.5061/dryad.f7b48.

## References

[1] Welcomme RL 1985 River fisheries. FAO Fisheries Technical Paper, no. 262. Rome, Italy: FAO.

[2] van Zalinge N, Degen P, Pongsri C, Nuov S, Jensen JG, Nguyen VH, Choulamany X 2004 The Mekong River System In *Proc. 2nd Int. Symp. on Management of Large Rivers for Fisheries, Phnom Penh, Cambodia* (ed. Welcomme RL, Petr T). Food and Agriculture Organization of the United Nations & The Mekong River Commission (http://www.fao.org/docrep/007/ad525e/ad525e0l.htm#bm21).

[3] de Graaf G 2003 Dynamics of floodplain fisheries in Bangladesh, results of 8 years fisheries monitoring in the Compartmentalization Pilot Project. Fish. Manage. Ecol. 10, 191–199. (doi:10.1046/j.1365-2400.2003.00339.x)

[4] Welcomme RL 1975 The fisheries ecology of African floodplains. CIFA Technical Paper, no. 3.

[5] Smolders AJP, Guerrero Hiza MA, van der Velde G, Roelofs JGM 2002 Dynamics of discharge, sediment transport, heavy metal pollution and Sábalo (*Prochilodus lineatus*) catches in the lower Pilcomayo river (Bolivia). River Res. Appl. 18, 415–427. (doi:10.1002/rra.690)

[6] Castello L, Isaac VJ, Thapa R 2015 Flood pulse effects on multispecies fishery yields in the Lower Amazon. R. Soc. open sci. 2, 150299 (doi:10.1098/rsos.150299) 26715994 10.1098/rsos.150299 PMC4680609

[7] Quiros R, Cuch S 1989 The fisheries and limnology of the lower Plata Basin. Spec. Publ. Can. J. Fish. Aquat. Sci. 106, 429–443.

[8] Risotto SP, Turner RE 1985 Annual fluctuation in abundance of the commercial fisheries of the Mississippi River and tributaries. North Am. J. Fish. Manage. 5, 557–574. (doi:10.1577/1548-8659(1985)5<557:AFIAOT>2.0.CO;2)

[9] Oliveira AG, Suzuki HI, Gomes LC, Agostinho AA 2015 Interspecific variation in migratory fish recruitment in the Upper Paraná River: effects of the duration and timing of floods. Environ. Biol. Fishes 98, 1327–1337. (doi:10.1007/s10641-014-0361-5)

[10] Junk WJ, Bayley PB, Sparks RE 1989 The flood pulse concept in river-floodplain systems. Spec. Publ. Can. J. Fish. Aquat. Sci. 106, 110–127.

[11] Bayley PB 1991 The flood pulse advantage and the restoration of river-floodplain systems. Regulated Rivers: Res. Manage. 6, 75–86. (doi:10.1002/rrr.3450060203)

[12] Kapetsky JM et al 1976 Fish populations in the floodplain lakes of the Magdalena River. Second report. Bogota, INDERENA-FAO, 30 p (mimeo).

[13] Cordiviola de Yuan E 1992 Fish populations of lentic environments of the Paraná River. Hydrobiologia 237, 159–173. (doi:10.1007/BF00005848)

[14] Wildlife Conservation Society. 2017 (http://amazonwaters.org/fish/curimata/).

[15] de Godoy MP 1959 Age, growth, sexual maturity, behaviour, migration, tagging and transplantation of the Curimbatá, Prochilodus scrofa Steindachner 1881, of the Mogi Guassu River, Sao Paulo State, Brasil. Anais Acad. bras. Cienc. 31, 447–477.

[16] Bonetto AA, Cordiviola de Yuan E, Pignalberi C, Oliveros O 1969 Ciclos y hidrológicos del Rio Paraná y las poblaciones de peces contenidas en las cuencas temporarias de su valle de inundación. Physis, Buenos Aires 29, 213–223.

[17] Bonetto AA, Pignalberi C, Cordiviola de Yuan E, Oliveros O 1971 Informaciones complementarias sobre migraciones de peces en la Cuenca del Plata. Buenos Aires 30, 505–520.

[18] Bayley PB 1973 Studies on the migratory characin, *Prochilodus platensis* Holmberg 1889 (Pisces, Characoidei) in the River Pilcomayo, South America. J. Fish Biol. 5, 25–40. (doi:10.1111/j.1095-8649.1973.tb04428.x)

[19] Loubens G, Panfili J 1995 Biology of *Prochilodus nigricans* (Teleostei: Prochilodontidae) in the Mamore basin (Bolivian Amazonia). Ichthyological exploration of freshwaters. Munchen 6, 17–32.

[20] Silva EA, Stewart DJ 2017 Reproduction, feeding and migration patterns of *Prochilodus nigricans* (Characiformes: Prochilodontidae) in northeastern Ecuador. Neotrop. Ichthyol. 15, e160171 (doi:10.1590/1982-0224-20160171)

[21] Rossi LM 1992 Evolución morfológica del aparato digestivo de postlarvas y prejuveniles de *Prochilodus lineatus* (Val., 1847) (Pisces, Curimatidae) y su relación con la dieta. Rev. Hydrobiol. Trop. 25, 159–167.

[22] Bayo V, Cordiviola de Yuan E 1996 Food assimilation of a neotropical riverine detritivorous fish, *Prochilodus lineatus*, studied by fatty acid composition (Pisces, Curimatidae). Hydrobiologia 330, 81–88. (doi:10.1007/BF00019997)

[23] Bayley PB 1983 Central Amazon fish populations: biomass, production and some dynamic characteristics. Doctoral dissertation, Dalhousie University, Nova Scotia, Canada.

[24] Bayley PB, Herendeen RA 2000 The efficiency of a seine net. Trans. Am. Fish. Soc. 129, 901–923. (doi:10.1577/1548-8659(2000)129<0901:TEOASN>2.3.CO;2)

[25] Petry P, Bayley PB, Markle DF 2003 Relationships between fish assemblages, macrophytes and environmental gradients in the Amazon River floodplain. J. Fish Biol. 63, 547–579. (doi:10.1046/j.1095-8649.2003.00169.x)

[26] Braga de Oliveira MI 1997 Determinação da idade e aspectos da dinâmica populacional do curimatã *Prochilodis nigricans* (Pisces: Prochilodontidae) da Amazônia Central. Masters dissertation Instituto Nacional de Pesquisas da Amazônia - Fundação Universidade do Amazonas.

[27] R Core Team. 2016 R: a language and environment for statistical computing. Vienna, Austria: R Foundation for Statistical Computing (https://www.R-project.org/).

[28] Petrere M Jr, Bayley PB, Paula GA 1991 Influential analysis of individual growth rates from five populations of *Prochilodus* spp. (Characoidei, Osteichthyes) in South America. Bol. Mus. Para. Emilio Goeldi, sér. Zool. 7, 125–142.

[29] Sippel SJ, Hamilton SK, Melack JM, Novo EMM 1998 Passive microwave observations of inundation area and the area/stage relation in the Amazon River floodplain. Int. J. Remote Sens. 19, 3055–3074. (doi:10.1080/014311698214181)

[30] Bayley PB 1988 Factors affecting growth rates of young tropical floodplain fishes: seasonality and density-dependence. Environ. Biol. Fishes 21, 127–142. (doi:10.1007/BF00004848)

[31] Batista VS, Petrere M Jr 2007 Spatial and temporal distribution of fishing resources exploited by the Manaus fishing fleet, Amazonas, Brazil. Braz. J. Biol. 67, 651–656. (doi:10.1590/S1519-69842007000400009) 18278316 10.1590/s1519-69842007000400009

[32] Fournier DA, Sibert JR, Majkowski J, Hampton J 1990 MULTIFAN a likelihood-based method for estimating growth parameters and age composition from multiple length frequency data sets illustrated using data for southern bluefin tuna (*Thunnus maccoyii*). Can. J. Fish. Aquat. Sci. 47, 301–317. (doi:10.1139/f90-032)

[33] Fournier DA, Hampton J, Sibert JR 1998 MULTIFAN-CL: a length-based, age-structured model for fisheries stock assessment, with application to South Pacific albacore, *Thunnus alalunga*. Can. J. Fish. Aquat. Sci. 55, 2105–2116. (doi:10.1139/f98-100)

[34] Kleiber P, Hampton J, Davies N, Hoyle S, Fournier DA 2014 MULTIFAN-CL User's Guide, September, 2014. (http://www.multifan-cl.org/).

[35] Batista VS, Barbosa WB 2008 Descarte de peixes na pesca comercial em Tefé, médio Solimões, Amazônia Central. Acta Sci. Biol. Sci. Maringá 30, 97–105.

[36] Venables WN, Ripley BD 2002 Modern applied statistics with S, 4th edn New York, NY: Springer.

[37] Dudley RG 1972 Biology of Tilapia on the Kafue floodplain, Zambia: predicted effects of the Kafue Gorge Dam. Doctoral dissertation, University of Idaho, Moscow, USA.

[38] Oliveira ACB, Soares MGM, Martinelli LA, Moreira MZ 2006 Carbon sources of fish in an Amazonian floodplain lake. Aquat. Sci. 68, 229–238. (doi:10.1007/s00027-006-0808-7)

[39] Junk WJ 1983 Ecology of swamps in the middle Amazon. In Mires, swamp, bog, fen, moor (ed. Gore AJP). pp 269–296. Amsterdam, The Netherlands: Elsevier.

[40] Arnesen AS, Silva TS, Hess LL, Novo EM, Rudorff CM, Chapman BD, McDonald KC 2013 Monitoring flood extent in the lower Amazon River floodplain using ALOS/PALSAR ScanSAR images. Remote Sens. Environ. 130, 51–61. (doi:10.1016/j.rse.2012.10.035)

[41] Hess LL, Melack JM, Affonso AG, Barbosa CCF, Gastil-Buhl M, Novo EMLM 2015 LBA-ECO LC-07 Wetland Extent Vegetation, and Inundation: Lowland Amazon Basin. Oak Ridge, TN: ORNL DAAC (http://dx.doi.org/10.3334/ORNLDAAC/1284).

[42] Welcomme RL 1979 The fisheries ecology of floodplain rivers. London, UK: Longman.

[43] Halls AS, Welcomme RL 2004 Dynamics of river fish populations in response to hydrological conditions: a simulation study. River Res. Appl. 20, 985–1000. (doi:10.1002/rra.804)

[44] Bayley PB 1995 Understanding large river: floodplain ecosystems. Bioscience 45, 153–158. (doi:10.2307/1312554)

