# Supplementary material for: Response of Prochilodus nigricans to flood pulse variation in the central Amazon
Source: R Soc Open Sci. 2018 Jun 13;5(6):172232. doi: 10.1098/rsos.172232 (PMC6030286; doi:10.1098/rsos.172232)
Supplement: Supplementary Material: Response of Prochilodus nigricans to flood pulse variation in the central Amazon [file rsos172232supp1.docx]

# Supplementary Material:

**Response of *Prochilodus nigricans* to flood pulse variation in the central Amazon**

Authors:

Peter B. Bayley ^1,*^, Leandro Castello ^2^, Vandick S. Batista^3^, Nidia N. Fabré^3^

^1^ Department of Fisheries and Wildlife, Oregon State University, Corvallis OR 97331. Present address: Rua Quinze 268 JD Guaraú, Peruibe-SP, CEP 11750-000, Brazil

^2^ Department of Fish & Wildlife Conservation, Virginia Polytechnic Institute and State University, Blacksburg, Virginia, USA.

^3^ Federal University of Alagoas. Institute of Biology Science and Health, Cidade Universitária, Maceió, Alagoas, Brazil

* Author for correspondence (piscesb@runbox.com)

**Manaus market data processing** (referred to in “The Manaus Fishery” in main text)

Fishing effort was defined as the product of (a) number of fishermen and (b) number of days fished per fisherman [29]. Both components were recorded for 83% of trip interviews. For our analysis, we needed totals for fishing effort by quarter and by 'fishery' (defined by gear type), therefore estimates for (a) and/or (b) were required for incompletely recorded trips. We did this by using the trip data with both effort components recorded (25,482 trips) to create predictive models, using log-transforms to normalise data. These models were included significant effects of region fished, and number of fishermen (a) or days fished (b) when (a) or (b) was recorded in incompletely recorded trips.

Having raw data or estimates for (a) and (b) for all trips, we then needed to estimate the prediction error of total effort estimates for each quarter-fishery to decide whether to include it in the analysis. This prediction error is expressed as the upper 95% confidence interval of the total effort as a percentage of the total effort from all trips in each quarter-fishery type. The 95% confidence interval is derived from the residual errors from the predictive models used in trips containing predictions. As would be expected, prediction error was zero for quarters containing no estimates, and increased with increasing proportions of estimated trips. The mean prediction error was 1.1% which was low compared with the Coefficient of Variation (CV = SD(Standard Deviation)*100/mean) of quarterly effort which was 77%. Errors less than 10% were accepted, which resulted in 1 of a total of 153 quarters among the three fishery types analysed being allocated as a missing value for effort.

When catch records indicated presence of *P. nigricans* but no weight data were available (i.e., 1019 trips, 3.3%), catch in weight was estimated based on a catch per effort (CPUE) model including significant effects of gear used and hydrological season. The prediction error (as defined above for effort) applied to quarterly data, averaged 1.2% while the CV of quarterly catch was 135%. Errors less than 10% were accepted, which resulted in 3 of the total of 153 quarters among the 3 gear types being allocated as missing values for catch.

In addition the assistant accounted for fishing boats that could not be interviewed. The market was open Monday through Saturday excepting Holidays. Catch and effort were adjusted each month for the proportion of market days and boats that were sampled. The proportion of market days sampled averaged 0.875 (range 0.28-1.0) and the proportion of boats interviewed averaged 0.828 (range 0.43-0.98).

Reference

29. Petrere M Jr (1978) Pesca e esforço de pesca no estado do Amazonas. I- Esforço e captura por unidade de esforço. Acta Amazonica 8(3): 439-454

**MULTIFAN-CL analysis** (referred to in “Analysis of fishery data” in main text)

Using a time series of catch, fishing effort and fish length distributions, MFCL minimises an objective function comprising the sum of negative log-likelihood functions of observed data and of Bayesian priors on various parameters. The length samples are regarded as random samples from an age-structured population. The proportions-at-age in the length frequencies are constrained by the catch equations to provide the mostly likely dynamic record of the age-structured population and the fishery. The widely-used catch equations (simplified for a given fishery, time, and age-class) are:

N_t+1_ = N_t_e^-Z^

where,

N_t+1_, N_t_ are cohort [age-class] population sizes at times t+1, t;

Z is total instantaneous mortality rate;

Z = F + M

where,

F and M are fishing and natural instantaneous mortality rates, respectively, and

C = (F/Z)[1-e^-Z^]N

where,

C is catch from a given age class, fishery and time period.

In addition, fishing effort, E, is related to F:

F = Eqs

where,

q is gear catchability,

s is gear selectivity.

Finally, a relative-age version of the von Bertalanffy growth model is applied:

FL_t_ = FL_1_ + (FL_n_ - FL_1_)(1 - exp(-K(t-1)))/(1 - exp(-K(n-1)))

where

FL_t_ = fork length at age t;

FL_1_ = mean fork length of the first age group;

FL_n_ = mean fork length of the n-th (oldest) age group;

K = growth rate coefficient;

n = no. of age-groups (yrs).

When fitting this function, length-at-age was assumed to vary and be normally distributed.

Alternative functional forms for critical variables are available, such as selectivity or catchability as functions of age or fish size. A plausible alternative to the default function is selected when the objective function is reduced because that implies a better overall fit. In nested models, such as comparing different numbers of age classes, the objective function changes according to Chi-squared and can therefore be tested [30]. The user can control the model’s parameter searches by means of Bayesian prior estimates in the form of ranges or means with variance. The user may have good independent evidence for the accuracy of a parameter, and therefore it could either be fixed, or input as a prior with a narrow distribution (i.e., high ‘penalty’ = low variance). However, if the model produces a value outside the expected distribution, a larger cost in likelihood is applied that increases the objective function. Conversely, the user may wish to allow the model to assess a parameter by inputting a wide distribution (low ‘penalty’ = high variance) for the prior. This is important if there is limited independent information available, or if such information is to be compared.

Each ‘fishery’ in MFCL is characterised by the gear used. Therefore, one or more fisheries that exploit a single stock can be modelled jointly, allowing for different catchability and selectivity characteristics. In our case, three fisheries are jointly modelled: lampara seine (fishery 1), gill net (fishery 2), and mixed lampara seine and gillnet (fishery 3) (Table 1). Fishing mortality rates, F, are estimated for each fishery, and combined for the whole fishery. In common with other fishery models, MFCL cannot estimate year-to-year changes in natural mortality, M, which would in any case require additional data. The model can estimate an average natural mortality rate, for which a prior of 0.45 and a low penalty of 3 (CV = 0.41) on a lognormal scale were used.

During its iterations, MFCL needs to account periodically for all parameter estimates throughout the time series. In our model, recruitment of *P. nigricans* was annual, while accounting dates were set at quarterly intervals (Jan.-Mar., April-June, etc.) because these respond most closely to typical hydrological seasons associated with the interaction of fish and fishers. For example, the highest yields occur when lampara seines exploit *P. nigricans* during the rapid drawdown period in July-September, followed by the second highest yield during the low-water period in September-December (Table 1). Conversely, the highest total gill net yield occurs during the low-water period, followed by that during the rising water period of January - March (Table 1). Therefore, catch, effort, and length-frequency data were pooled in those intervals for each fishery and each year. Catchability by fishery was also allowed to vary seasonally corresponding to the four quarterly periods.

The recommended option of estimating age-specific selectivity via a length-based approach (in which the overlapping lengths of different age classes are accounted for) was used for each fishery. The functional form of selectivity depended on the gear. For lampara seine (fishery 1) a logistic model, in which selectivity increases with age class, provided the best fit. Regarding gillnets (fishery 2), size-selectivity by mesh size indicated a large variation for a morphologically similar species, *Gasterotomus latior*, [33] in which length ranges caught by mesh sizes (bar = 4, 5, 6, and 8 cm) were 7.5, 9.5, 8 and 6.5 cm, respectively. When Barthem’s [33] selectivity model was applied to the distribution of mesh sizes from the gillnet fishery (6731 samples, range 2-36 cm), a unimodal curve was obtained. Subsequently a cubic spline selectivity function for a single mode was used to derive appropriate parameters for *P. nigrican*s in fishery 2. The combined fishery 3 was assigned the default freeform model by age class.

References

30. Fournier DA, Sibert JR, Majkowski J, Hampton J (1990) MULTIFAN: a likelihood-based method for estimating growth parameters and age composition from multiple length frequency data sets illustrated using data for southern bluefin tuna (*Thunnus maccoyii*). Canadian Journal of Fisheries and Aquatic Sciences  47: 301-317

33. Barthem RB (1984 ) Pesca experimental e selectividade de redes de espera para espécies de peixes amazônicos. Boletim do Museu Paraense Emílio Goeldi Zoologia 1(1): 57-88

**Supplementary Table S1.** Mean selectivity estimates by age class and fishery

| Fishery    Age class: Age1 Age2 Age3 Age4+  fishery 1 0.043 0.348 0.873 1.000  fishery 2 0.000 0.0014 1.000 1.000  fishery 3 0.037 0.593 1.000 1.000 |
| --- |
